# Case report: Refractory focal motor seizure associated with cerebrospinal fluid neurochondrin antibody

**DOI:** 10.3389/fimmu.2024.1459119

**Published:** 2024-09-23

**Authors:** Rowaid Ahmad, Yumeng Huang, Peter R. Wang, Todd Masel, Xiangping Li

**Affiliations:** ^1^ Department of Neurology, University of Texas Medical Branch, Galveston, TX, United States; ^2^ School of Medicine, University of Texas Medical Branch, Galveston, TX, United States

**Keywords:** autoimmune epilepsy, neurochondrin antibody, norbin, autoimmune cerebellar ataxia, autoimmune encephalitis

## Abstract

**Background:**

Focal onset seizures, characterized by localized neuronal hyperexcitability in the brain, can be related to various structural, immune, genetic, or metabolic abnormalities. Autoimmune epilepsies are increasingly recognized. Neurochondrin antibody has been reported in a variety of rare autoimmune neurological disorders. This article aims to highlight the relevance of anti-neurochondrin in autoimmune epilepsy.

**Methods:**

This is a case presentation and literature review of autoimmune epilepsy associated with anti-neurochondrin antibody.

**Case presentation:**

A 26-year-old African American right-handed man with a history of Sjogren’s syndrome presented with near constant, rhythmic left-sided facial twitching movements, and one episode of generalized tonic clonic seizure. Magnetic resonance imaging (MRI) of the brain revealed borderline low volume right hippocampus. Cerebrospinal fluid (CSF) studies yielded elevated protein and mild lymphocytic pleocytosis. Antibody Prevalence in Epilepsy 2 (APE2) score was 6, and autoimmune workup was initiated. Anti-neurochondrin antibody returned positive in the CSF autoimmune encephalitis panel with a titer of 1:512 (Mayo Clinic TEST ID: ENC2). Seizures remained refractory to anti-seizure medications including divalproex, lacosamide, and oxcarbazepine. Immunotherapy with methylprednisolone and immunoglobulin improved his epileptic seizures.

**Conclusion:**

This is the first reported case of refractory autoimmune epilepsy with positive CSF anti-neurochondrin antibody. This study contributes to the body of evidence supporting the role of neurochondrin antibody in epilepsy. Considering autoimmune testing in individuals with seizures having APE2 score > 4 can aid in timely diagnosis of immune-mediated epilepsy and initiation of immunotherapy, which can result in favorable clinical outcomes. Diagnosis of autoimmune epilepsy, in most cases, is based on clinical characteristics, MRI results, and CSF findings. In addition to the traditional antibody panel for autoimmune encephalitis, some novel antibodies, such as anti-neurochondrin, should also be considered.

## Introduction

Autoimmune epilepsy has been increasingly recognized in the spectrum of neurological disorders in the last decade, defined as a group of epilepsies with evidence of immune- mediated inflammatory disease in the central nervous system. It has been reported to be present in up to 20% of patients with epilepsy of unknown etiology (1). Among patients with epilepsy, approximately one-third of them do not respond to antiseizure medication (ASM) treatment (2). However, immunomodulatory treatment is increasingly seen as a treatment option in some refractory autoimmune epilepsy cases. In recent decades, numerous neural autoantibodies have been discovered among patients with epilepsy. From the standpoint of pathogenesis, autoantibodies targeting cell surface or intracellular antigens were shown to induce epilepsy via different mechanisms. For example, cell surface antibodies including leucine-rich glioma-inactivated protein 1 (LGI1) and N-ethyl-D-aspartate receptor (NMDA-R) have a direct pathogenic role, whereas, in intracellularly present autoantigens such as glutamic acid decarboxylase 65 (GAD65), anti-Hu, or anti-Yo, neuronal injury is caused by T-cell cytotoxic response (3, 4). The neuronal injury induced by autoantibodies can cause varied presentations, including but not limited to memory decline, psychiatric symptoms, seizures, and other neurological deficits. It is essential that autoimmune etiology should be considered in the initial differential diagnosis of new onset intractable epilepsy. Early treatment, as well as maintenance immunotherapy where appropriate, facilitates an optimal outcome for the patient (5).

In recent years, neurochondrin antibody has been reported in some autoimmune encephalitis cases since 2016 (6). Based on studies, neurochondrin is richly expressed in cerebellum, brainstem, amygdala, and hippocampus. So far, neurochondrin autoimmunity is reported in autoimmune encephalitis, rapidly progressive rhomboencephalitis with cerebellar ataxia and brainstem signs, cerebellar degeneration, chorea, and Alzheimer’s disease (3, 5–7).

This article aims to report the first case of autoimmune epilepsy with cerebrospinal fluid (CSF)–positive anti-neurochondrin antibody, presenting with refractory focal motor seizures with significant weight loss, concurrent neuropsychiatric disturbance, and Sjogren’s syndrome. Also, a literature review on neurochondrin antibody was conducted and summarized in this article. The distinctive phenotype in our case has not been reported in neurochondrin antibody–related neurological disorders and will contribute to the pool of literature to understand this rare entity.

## Methods/literature search

Literature research through academic databases—PubMed, Ovid, and Scopus—was used for systematic searching. Keywords applied for searching are “neurochondrin” or “anti-neurochondrin antibody.” Forty-nine publications were identified from this topic, including neurochondrin-related basic science literature and case reports. After searching PubMed, Ovid, and Scopus, 10 case/series case reports have been found with neurochondrin positive findings (PubMed, n = 6; Ovid, n = 6; and Scopus, n = 10).

## Case presentation

A 26-year-old man with past medical history of Sjogren’s syndrome presented to the emergency room with new onset left-sided facial twitching for 2 weeks. The patient reported these episodes as twitching of lips, which have been constant in nature both during wakefulness and sleep but occurring more during the day than nighttime. These episodes have been getting progressively worse. He also reported a 1- to 2-min episode of generalized tonic-clonic seizure characterized by bilateral limbs shaking and stiffening. This episode was witnessed by his mother and decided to bring him to the emergency department.The patient also reports a 60-pound weight loss over the past 6 months, along with occasional night sweats and subjective fevers. He had increased anxiety and became more irritable according to his family. His social history was remarkable for occupation as a waiter and regular use of marijuana although he denied tobacco or alcohol use.

During examination, he was alert and oriented by the time he arrived to the hospital. There was noticeable left-sided facial twitching, including the lip, occurring in the range of 10 to 30 times a minute. Frequency of this movement decreased during rest or sleep and increased during activity, especially talking. Otherwise, his neurologic exam was unremarkable. There was no formal cognitive exam conducted.

**Figure 1:**
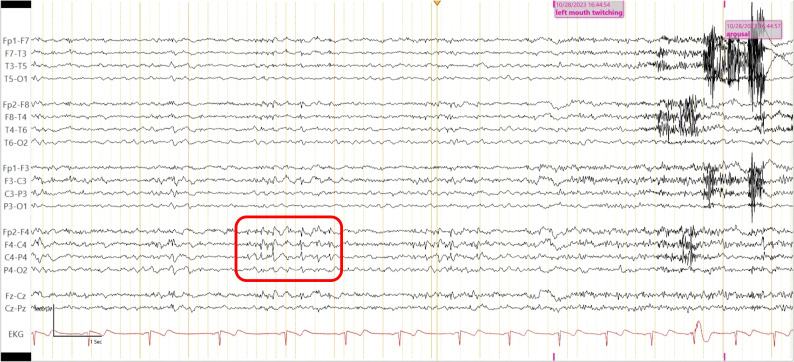
Routine EEG shows epileptiform spikes and sharp wave over the right fronto- central region (red square), maximally at electrode F4-C4.

**Figure 2:**
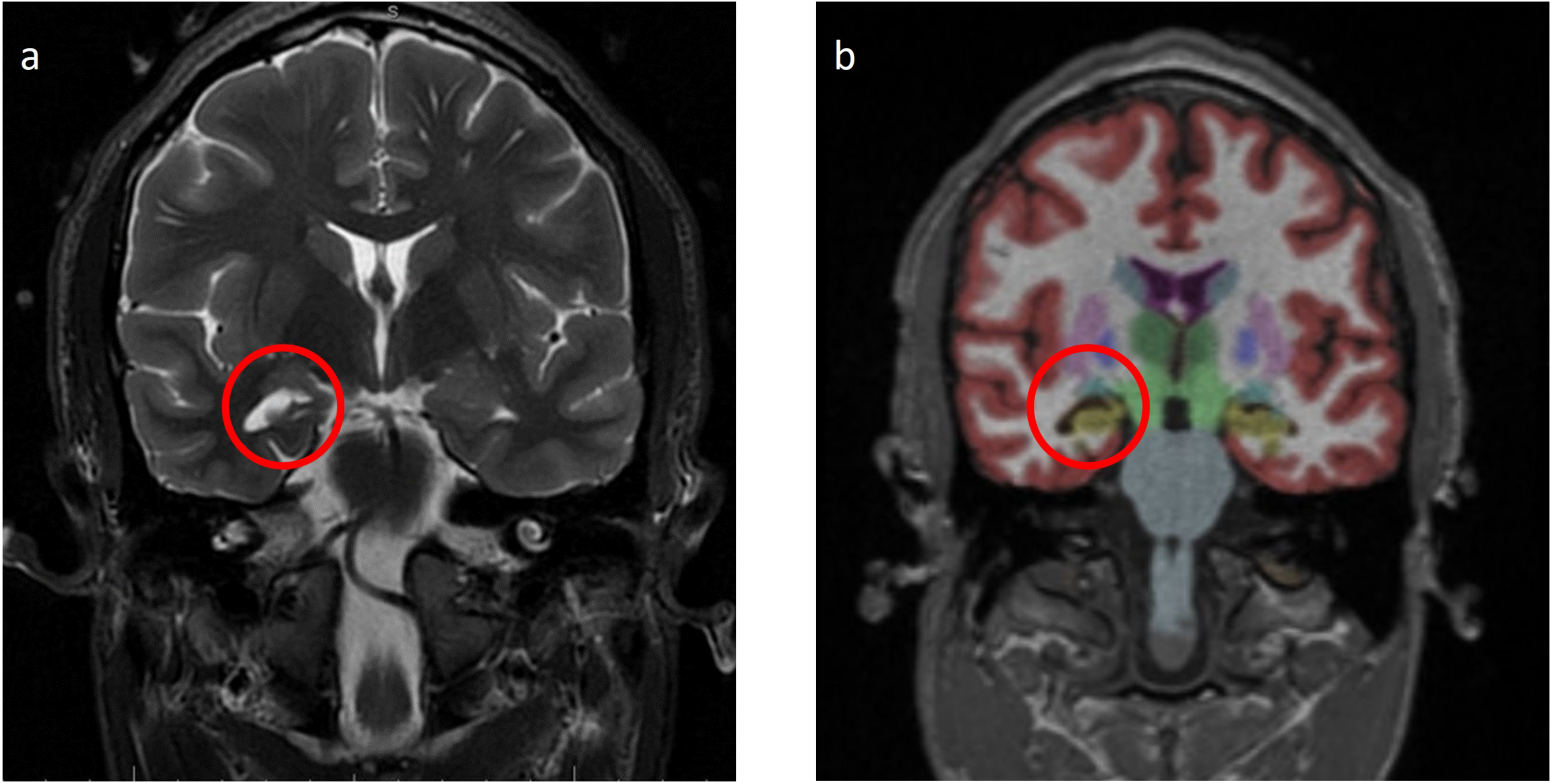
**(A)** Magnetic resonance imaging (MRI) Brain W/WO Contrast T2 Fluid Attenuated Inversion Recovery (FLAIR): low right hippocampus volume (red circle). **(B)** MRI Brain with NeuroQuant a few weeks after the initial admission shows the low right hippocampus volume (fifth percentile, yellow area in the red circle).

Laboratory tests, including complete blood count, complete metabolic panel, thyroid-stimulating hormone, and Free T4, were within reference ranges. He had an established diagnosis of Sjogren’s syndrome and previously reported antinuclear antibody (ANA)–positive titer 1:160 (reference range, <1:80) and anti-Sjogren’s syndrome antibody (Anti-Ro/SSA). There was elevated creatine kinase (CK) at 246 U/L (reference range, 22–198 U/L), and urine drug screen was positive for marijuana. HIV, syphilis, and QUANTIFERON TB assay were negative. His recent weight loss and mood changes were concerning for underlying malignancy. Thus, a whole-body CT scan and scrotal ultrasound were obtained, which were unremarkable. A routine electroencephalogram (EEG) showed occasional epileptiform spikes and sharp slow wave over the right fronto-central region (**Figure 1**). Associated video showed near constant left-sided facial twitching during day and night. However, EEG findings did not fulfill criteria of Brief Potentially Ictal Rhythmic Discharges (BiRDS) nor were any electrographic seizures seen. Magnetic resonance imaging (MRI) of the brain revealed low right hippocampus volume (**Figure 2A**). CSF showed elevated protein at 125 mg/dL and lymphocytic pleocytosis at 7/µL. CSF meningitis/encephalitis panel by polymerase chain reaction was negative. CSF cytology was normal, and serum and urine electrophoresis showed that the total protein was at 6.1 g/dL and a normal distribution of immunoglobulins. The Antibody Prevalence in Epilepsy and Encephalopathy 2 (APE2) score was calculated to be 6 (new onset seizure, +1; refractory seizure, +2; neuropsychiatric changes, +1; and CSF abnormality, +2), increasing suspicion for a neural antibody-mediated process. Thus, Autoimmune/paraneoplastic Serum Panel (ARUP 3006050) and Autoimmune/paraneoplastic encephalitis, CSF (Mayo ID: ENC2), were sent for further analysis for autoimmune and paraneoplastic etiologies and pending at this point.

The patient was treated with levetiracetam, valproate, and divalproex extended release (ER). Given his stability in symptoms and at the patient’s preference, he was discharged on divalproex ER 750 mg twice a day (BID) and intranasal midazolam of 5 mg per spray.

A few weeks after his initial admission, the patient presented to the ED after a witnessed tonic-clonic seizure and LOC for about 10 min. The patient had postictal confusion as well but without bowel or bladder incontinence. Head imaging was unremarkable for any acute abnormalities. Seizure secondary to medication noncompliance was ruled out after valproate levels were found to be at 10.9 µg/ml. Thus, lacosamide was added to his ASM regimen. The physical exam showed persistent left-sided cheek and lower lip twitching. The Autoimmune/paraneoplastic Serum Panel (ARUP Laboratories: 3006050) returned negative (**Table 1**). MRI brain with and without contrast with NeuroQuant and seizure protocol (**Figure 2B**) showed a borderline low right hippocampus volume and minimal white matter hyperintensities in T2 Fluid-Attenuated Inversion Recovery (FLAIR). PET/CT imaging showed diffusely increased activity of both parotid glands and submandibular glands in keeping with Sjogren’s syndrome, otherwise no abnormal activity of the chest, abdomen, or pelvis.

**Table 1:** Blood and CSF neuro-immunological testing.

Neuro-immunologic testing (serologically)	Results	Reference	Neuro-immunologic testing (CSF)	Results	Reference
NMDA-R	<1:10	<1:10	AMPA-R	Negative	Negative
CASPR-2	<1:10	<1:10	Amphiphysin	Negative	Negative
AMPA-R	<1:10	<1:10	AGNA-1	Negative	Negative
GABA-BR	<1:10	<1:10	ANNA-1	Negative	Negative
MOG	<1:10	<1:10	ANNA-2	Negative	Negative
DPPX	<1:10	<1:10	ANNA-3	Negative	Negative
GABA-AR	<1:10	<1:10	CASPR2	Negative	Negative
IgLON5	<1:10	<1:10	CRMP-5	Negative	Negative
mGluR1	<1:10	<1:10	DPPX	Negative	Negative
Voltage-Gated Potassium Channel	15 pmol/L	0 - 31	GABA-BR	Negative	Negative
Glutamic Acid Decarboxylase	<5.0 Iu/mL	0.0-5.0	GAD65	0.00 nmol/L	≤0.02
			GFAP	Negative	Negative
			IgLON5	Negative	Negative
			LGI-1	Negative	Negative
			mGluR1	Negative	Negative
			**Neurochondrin**	**1:512**	**<1:2**
			NIF	Negative	Negative
			NMDA-R	Negative	Negative
			PCA-Tr	Negative	Negative
			PCA-1	Negative	Negative
			PCA-2	Negative	Negative
			Septin-7	Negative	Negative

Abnormal values are printed in bold. AMPA-R, alpha-amino-3-hydroxy-5- methyl-4-isoxazolepropionic acid receptor; AGNA-1, anti-glial nuclear antibody–1; ANNA-1/2/3, anti- neuronal nuclear antibody–1/2/3; Anti-IgLON5, anti–immunoglobulin-like cell adhesion molecule-5; anti-mGluR1, anti-metabotropic glutamate receptor–1; CASPR-2, contactin-associated protein–2; CRMP-5, collapsin response mediator protein–5; CSF, cerebrospinal fluid; DPPX, dipeptidyl-peptidase-like protein–6; GAD, glutamate decarboxylase; GABA-A/BR, γ- aminobutyric acid-A/B receptor; LGI-1, leucine-rich glioma-inactivated–1; NMDA-R, N-methyl-d-aspartate receptor; PCA-Tr/1/2, Purkinje cell cytoplasmic antibody type–Tr/1/2; GFAP, glial fibrillary acidic protein; NIF, neuronal intermediate filament; MOG, myelin oligodendrocyte glycoprotein.

Epilepsy monitoring unit (EMU) testing was recommended, and the patient followed up with his neurologist after the discharge. He self-discontinued lacosamide due to possible adverse effects of tongue swelling and lack of improvement to his facial twitches. He underwent 3 days of EMU testing. Valproate level was at 114 µg/mL on admission. He remained fully awake and alert but exhibited nearly continuous left facial twitching during sleep and wakefulness with fluctuation in intensity and duration. The video EEG showed left facial twitching, without electrographic correlates on scalp EEG. However, this could be seen in epileptic seizures with an epileptogenic focus too deep and/or small to have an ictal correlate on the scalp EEG. The patient also received one dose of intravenous (IV) lorazepam with temporary improvement in his facial twitching although they recurred at a reduced intensity. Interictally, there were occasional epileptiform spikes and sharp waves as well as focal delta slowing in the right hemisphere, indicative of focal cortical irritability and focal cerebral dysfunction over that region. These findings are consistent with the clinical diagnosis of focal aware seizures.

At this time, his autoimmune/paraneoplastic encephalitis, CSF (Mayo ID: ENC2), was updated to show positive anti-neurochondrin antibodies at a titer of 1:512 (**Table 1**). The patient was directly admitted for immunosuppressive treatment. He continued to have constant left facial twitching with valproate free level at 20.0 µg/mL on admission. He received 5 days of methylprednisolone at 1 g IV and 5 days of intravenous immunoglobulin (IVIG). Pt experienced some nephrotoxicity due to the IVIG on day 3, and treatment was continued at a reduced flow rate. Patient responded well to treatment and endorsed improvement in facial twitching and speech. Oxcarbazepine 300 mg BID was started in addition to the divalproex at 1500 mg BID, and the patient was instructed to follow-up with his neurologist for maintenance IVIG as outpatient given his insurance was not accepted at our institution.

Approximately 3 months after discharge, the patient’s mother was contacted over the phone, and she reported an improvement in frequency and intensity of facial myoclonus while receiving IVIG therapy. Unfortunately, the patient had to discontinue IVIG treatment after three sessions and could not follow up in clinic afterward due to lack of medical insurance.

## Discussion

Here, we report the first case of neurochondrin antibody–associated refractory focal motor seizures with concurrent psychiatric symptoms. Neurochondrin is a newly discovered neuronal target antigen first reported by Miske et al. in 2016 in three patients who presented with autoimmune cerebellar degeneration (6). Per review of literature, only 19 cases with cerebellar ataxia and positive anti-neurochondrin antibody testing have been reported (8). A case series was conducted at Mayo Clinic by Shelly et al. to describe the neurological spectrum of patients with neurochondrin-immunoglobulin G (IgG) positivity in serum and CSF. Of the seven patients with available clinical data, six presented with rapidly progressive cerebellar ataxia, brainstem signs, or both, whereas one had unexplained psychiatric symptoms. Among those, 5 of the patients were women (3). There is only one case describing Alzheimer’s dementia associated with anti-neurochondrin antibody found in the CSF (5). Additionally, one case of chorea in a child was reported to be associated with anti-neurochondrin antibody in the serum (9). Its association with seizures has not been reported in the literature thus far. The role of an underlying autoimmune disease in the pathogenesis of autoimmune epilepsy cannot be excluded either. Shelly et al. have reported one case of anti-neurochondrin positive autoimmune encephalitis with concurrent Sjogren’s disease (3). Anti-Ro/SSA antibodies are among the commonly detected antibodies in Sjogren’s syndrome and systemic lupus erythematosis (SLE) and are found to be associated with pathogenesis of autoimmune diseases. Of note, our patient is ANA positive and anti-Ro/SSA positive, and it cannot exclude the potential role of autoimmune antibodies in our case.

Neurochondrin is a leucine-rich antigen located in the brain, bone, and chondral tissues. Immunoreactivity pattern carried out in mice brain determined the antigen presence in the cerebellar Purkinje cells, brainstem, lateral parts of the central amygdala nuclei, and hippocampal pyramidal cells. It is an intracellular neuronal antigen which activates B- cells but requires additional CD8+ and CD4+ cytotoxic T-cell response for cellular death. This pathogenesis was endorsed by immune phenotyping which revealed intrathecal accumulation and activation of B and T cell receptor during acute phase of disease (6).

Neurochondrin (NCDN) is also known as norbin (neurite-outgrowth–related protein) in animal models (e.g., *Rattus norvegicus*) which is a cytoplasmic protein encoded by the *Ncdn* gene (e.g., in *Rattus norvegicus*). The exact role of neurochondrin in the epileptogenesis is unclear, but, in an animal study conducted by Dateki et al., neurochondrin negatively regulates calmodulin-dependent protein kinase II (CaMKII) phosphorylation and epileptic seizures were observed in mice after preferential elimination of neurochondrin gene expression in the nervous system by the conditional knock-out strategy (10). In the subsequent animal studies, Wang et al. also reported norbin as an important endogenous regulator of metabotropic glutamate receptor 5 (mGluR5) signaling in norbin conditional knockout mice (11).

In cases of neurochondrin positive vestibulocerebellar ataxia, CSF findings were found to be consistent with signs of inflammation: elevated protein, lymphocytosis, and increased IgG index (6, 9). MRI brain in these cases has shown cerebellar degeneration, which is in coherence with the antigen’s presence in cerebellum (Purkinje cells and diffusely in molecular layer). Mesial temporal lobe as a seizure focus is well known in cases of hippocampal sclerosis.

Currently, there are no strict guidelines for the diagnosis of autoimmune epilepsy. The typical work up in cases with epilepsy of unknown etiology involves MRI brain with and without gadolinium contrast, EEG, and CSF studies. In addition, antibody studies for autoimmune and paraneoplastic origin should be carried out in both serum and CSF. MRI brain may be normal during early disease course but can demonstrate T2 FLAIR hyperintensities in the cortical or subcortical regions in addition to unilateral atrophic changes. Based on the antigenic preference, various patterns of cortical atrophy can be observed. For instance, LGI1-associated encephalitis has shown hippocampal atrophy in follow-up MRI brain (12). A possible autoimmune etiology for seizures may be considered in the presence of continuous slow waves, frontal intermittent rhythmic delta activity, and delta brush pattern in the EEG. During the progression of disease, like the case at hand, inter-ictal epileptiform or focal ictal rhythmic discharges can also develop (13). CSF may be normal or may demonstrate elevated CSF protein and lymphocytosis like our case (protein at 125 mg/dL and lymphocytes at 7/µL). In our patient, we obtained autoimmune and paraneoplastic encephalopathy panel in CSF (Mayo ID # ENC2), which showed neurochondrin positive titer at 1:512. As a limitation of our study, we lack serum studies of neurochondrin antibody since the studies were sent to a different laboratory (ARUP), which does not include this antibody as part of the panel.

Diagnostic criterion to determine autoimmune-associated epilepsy (AAE) has been proposed by Dubey et al. using APE2 score, neural-specific antibody status, and trial of immunotherapy. It suggests that all patients with epilepsy of unknown etiology and APE2 score ≥ 4 should undergo autoantibody evaluation. If the neural specific antibody detected is clinically associated with autoimmune epilepsy, then the case would meet criteria for “definite autoimmune epilepsy.” In our patient, APE2 score was at 6 (new onset seizure, +1; refractory seizure, +2; neuropsychiatric changes, +1; and CSF, abnormality, +2) (14). With the currently practiced diagnostic criteria and our existing knowledge of neurochondrin presence in neural tissue, this is a novel case of neurochondrin-related AAE.

Therapeutic strategies in AAE are targeted toward immunoregulation and managing seizures. First-line immunotherapeutic agents considered for such patients are high- dose intravenous methylprednisolone (IVMP), IVIG, or plasma exchange. Second-line agents including rituximab, cyclophosphamide, mycophenolate, azathioprine, and tocilizumab are added for refractory seizure cases or as maintenance therapy to avoid relapses (4). ASMs with considerable benefit in such cases are sodium channel-blocking drugs such as carbamazepine, lacosamide (in combination with phenytoin), or oxcarbazepine (15). Our patient responded to initial IVMP and IVIG treatment along with oxcarbazepine that provided improved seizure control. He continued to improve on the frequency and intensity of his focal motor seizures on follow-up maintenance IVIG sessions.

## Conclusion

The neurochondrin-associated neurological disorder is rare and its principal clinical manifestations observed so far are cerebellar ataxia, rhombencephalitis, and hyperkinetic movements such as chorea. Neurochondrin antibody–associated autoimmune epilepsy has not been previously reported in the literature. Autoimmune testing in individuals with seizure of unknown etiology and APE2 score ≥ 4 can help in timely diagnosis and management of underlying autoimmunity. Early treatment with immunotherapy and maintenance where appropriate can assure a more favorable outcome for the patients. Our case highlights the importance of seeking autoimmune testing in individuals with seizures of unknown etiology and inclusion of neurochondrin antibody testing in the standard autoimmune epilepsy workup. We emphasize the importance of maintaining a high degree of suspicion for autoimmune encephalopathy and low threshold for testing especially when a patient has new-onset refractory seizures. Further studies are warranted to help expand the clinical spectrum and reveal the pathophysiology of the neurochondrin-associated neurological disorders.

## Data Availability

The datasets presented in this article are not readily available because of ethical and privacy restrictions. Requests to access the datasets should be directed to the corresponding author/s.

## References

[1] Cabezudo-García P Mena-Vázquez N Ciano-Petersen NL García-Martín G Estivill-Torrús G Serrano-Castro PJ. Prevalence of neural autoantibodies in epilepsy of unknown etiology: systematic review and meta- analysis. Brain Sci. (2021) 11:392. doi: 10.3390/brainsci11030392 33808902 PMC8003737

[2] Flammer J Neziraj T Rüegg S Pröbstel AK. Immune mechanisms in epileptogenesis: update on diagnosis and treatment of autoimmune epilepsy syndromes. Drugs. (2023) 83:135–58. doi: 10.1007/s40265-022-01826-9 PMC9875200 36696027

[3] Shelly S Kryzer TJ Komorowski L Miske R Anderson MD Flanagan EP. Neurochondrin neurological autoimmunity. Neurol Neuroimmunol Neuroinflamm. (2019) 6:e612. doi: 10.1212/NXI.0000000000000612 31511329 PMC6745726

[4] Husari KS Dubey D. Autoimmune epilepsy. Neurotherapeutics: J Am Soc Exp Neurother. (2019) 16:685–702. doi: 10.1007/s13311-019-00750-3 PMC6694338 31240596

[5] Hansen N Malchow B Teegen B Wiltfang J Bartels C. Case report: alzheimer’s dementia associated with cerebrospinal fluid neurochondrin autoantibodies. Front Neurol. (2022) 13:879009. doi: 10.3389/fneur.2022.879009 35785337 PMC9243764

[6] Miske R Gross CC Scharf M Golombeck KS Hartwig M Bhatia U. Neurochondrin is a neuronal target antigen in autoimmune cerebellar degeneration. Neurol Neuroimmunol Neuroinflamm. (2017) 4:e307. doi: 10.1212/NXI.0000000000000307 27957508 PMC5141526

[7] Weihua Z Haitao R Fang F Xunzhe Y Jing W Hongzhi G. Neurochondrin antibody serum positivity in three cases of autoimmune cerebellar ataxia. Cerebellum (London England). (2019) 18:1137–42. doi: 10.1007/s12311-019-01048-y 31179511

[8] Schwarzwald A Salmen A León Betancourt AX Diem L Hammer H Radojewski P. Anti-neurochondrin antibody as a biomarker in primary autoimmune cerebellar ataxia—A case report and review of the literature. Eur J Neurol. (2023) 30:1135–47. doi: 10.1111/ene.15648 36437687

[9] Rommel FR Miske R Stöcker W Arneth B Neubauer BA Hahn A. Chorea minor associated with anti-neurochondrin autoantibodies. Neuropediatrics. (2017) 48:482–3. doi: 10.1055/s-0037-1606371 28962041

[10] Dateki M Horii T Kasuya Y Mochizuki R Nagao Y Ishida J. Neurochondrin negatively regulates CaMKII phosphorylation, and nervous system-specific gene disruption results in epileptic seizure. J Biol Chem. (2005) 280:20503–8. doi: 10.1074/jbc.M414033200 15790563

[11] Wang H Westin L Nong Y Birnbaum S Bendor J Brismar H. Norbin is an endogenous regulator of metabotropic glutamate receptor 5 signaling. Science. (2009) 326:1554–1557. doi: 10.1126/science.1178496 20007903 PMC2796550

[12] Wang M Cao X Liu Q Ma W Guo X Liu X. Clinical features of limbic encephalitis with LGI1 antibody. Neuropsychiatr Dis Treat. (2017) 13:1589–96. doi: 10.2147/NDT.S136723 PMC5481279 28670128

[13] Baysal-Kirac L Tuzun E Altindag E Ekizoglu E Kinay D Bilgic B. Are there any specific EEG findings in autoimmune epilepsies? Clin EEG Neurosci. (2016) 47:224–34. doi: 10.1177/1550059415595907 26240088

[14] Dubey D Pittock SJ McKeon A. Antibody Prevalence in Epilepsy and Encephalopathy score: Increased specificity and applicability. Epilepsia. (2019) 60:367–9. doi: 10.1111/epi.14649 30727035

[15] Feyissa AM López Chiriboga AS Britton JW. Antiepileptic drug therapy in patients with autoimmune epilepsy. Neurol Neuroimmunol Neuroinflamm. (2017) 4:e353. doi: 10.1212/NXI.0000000000000353 28680914 PMC5489139

